# A study protocol for a randomized controlled trial evaluating the impact of adding community health worker coaching calls and healthy grocery bag deliveries to a Meals on Wheels home-delivered meal program for homebound older adults in Rhode Island

**DOI:** 10.1186/s12889-025-24080-6

**Published:** 2025-10-01

**Authors:** Isabelli L. Costa da Silva, Kim M. Gans, Kali S. Thomas, Roee Gutman, Brie Tyler, Snehaa Ray, Meghan Grady, Shana DeFelice, Maya Hussein, Amelia Lusi, Caitlin E. Caspi

**Affiliations:** 1 Department of Allied Health Sciences, University of Connecticut, Storrs, CT USA; 2 Department of Human Development and Family Sciences, University of Connecticut, Storrs, CT USA; 3 Department of Behavioral and Social Sciences, Brown University School of Public Health, Providence, RI USA; 4 School of Nursing, Johns Hopkins University, Baltimore, MD USA; 5 Department of Biostatistics, Brown University School of Public Health, Providence, RI USA; 6 UConn Rudd Center for Food Policy and Health, University of Connecticut, Storrs, CT USA; 7 Department of Nutritional Sciences, University of Connecticut, Storrs, CT USA; 8 Meals on Wheels of Rhode Island, Inc, Providence, RI USA

**Keywords:** Aging, Nutrition, Community-based participatory research

## Abstract

**Background:**

Home-delivered meal programs (HDMP), such as Meals on Wheels, offer nutritious meals for homebound older adults experiencing nutritional risk. Despite receiving meals, participants may still have difficulty achieving nutritional goals, overcoming social isolation, and addressing other health issues. We aim to evaluate the impact of adding enhancements to traditional HDMP on improving diet quality, food and nutrition security, loneliness, and health-related quality of life among older adults in a randomized controlled trial.

**Methods:**

Homebound older adults at nutritional risk and participating in the Meals on Wheels of Rhode Island, Inc. (MOWRI) HDMP are randomized to receive either a usual care control group of the traditional HDMP (5 meals delivered per week) or the enhanced program (Meals+), which includes four Community Health Worker (CHW) coaching calls using motivational interviewing, and delivery of three healthful grocery bags during 12 weeks, in addition to the traditional HDMP. The primary outcome is diet quality measured by the validated Dietary Screening Tool (DST). Food and nutrition security, loneliness, and health-related quality of life are secondary outcomes assessed by validated measures. In the 12-week follow-up call, CHWs also ask participants about utilization and satisfaction with the intervention. The usual care group receives coaching from CHWs to connect them to community resources in this follow-up call. Study procedures were tested in a pilot randomized controlled trial (*n* = 12), resulting in modifications to the study protocol.

**Discussion:**

Enhancements such as CHW calls and grocery bags can help HDMP target food access, social and health interventions for older adults. These enhanced HDMP have the potential to be sustained and replicated nationwide.

**Trial registration:**

Number NCT06401694; Start date: 2024-06-20.

**Supplementary Information:**

The online version contains supplementary material available at 10.1186/s12889-025-24080-6.

## Background

Food insecurity is defined as a lack of access to enough food to maintain an active and healthy life [1]. In 2023, 13.5% of households in the United States were food insecure [1]. This number was higher for low-income older adults living alone, with 29% of those living below the poverty line experiencing food insecurity [1]. Among older adults, food insecurity is associated with poorer diet quality and higher rates of chronic disease [2, 3]. Some older adults experiencing food insecurity must choose between spending money on food or medication, which further compromises their health [4].

It is common for older adults to suffer from numerous nutritional deficiencies and have difficulty reaching their daily nutrient intake goals [5]. A longitudinal study among older adults observed that those with food insecurity had significantly lower Healthy Eating Index (HEI) scores, which implies lower diet quality and higher prevalence of nutritional deficiencies [6, 7]. In this sense, many older adults have difficulties in managing their own nutritional needs and could benefit from food programs, such as home-delivered meal programs [4, 8].

Home-delivered meal programs (HDMP) are among the tools used to combat food insecurity among older adults and other at-risk groups [9–12]. Community-based HDMP delivers healthy meals to older adults to improve diet quality and promote independent living, while also providing informal daily well-being checks. This can result in reduced hospitalizations, shorter hospital stays, fewer readmissions, and ultimately, lower healthcare costs [11, 13]. Systematic reviews of observational and intervention studies demonstrate that HDMP may also impact older adults’ quality of life, by reducing frailty, loneliness, and risk factors that exacerbate chronic diseases [10, 14]. The psychosocial benefits of HDMP derive from the interface between the community-based workforce and older adults in their homes, connecting them with food and health services [15–18]. Many older adults have few social ties, which makes the work of HDMP paid and volunteer drivers essential for social interaction through daily informal well-being checks [18, 19].

Despite the benefits, 70% of HDMP recipients do not meet their daily nutritional needs [9]. Some evidence suggests that increasing the number of meals delivered could strengthen the impact of HDMP on energy and protein intake, participants’ satisfaction, and overall diet quality [10, 11]. Evidence from multiple studies shows that providing more than one meal per day, providing meals rich in protein, and nutritional counseling is associated with higher calorie and protein intake among older adults [9, 10, 12].

Similarly, an intervention with low-income older adults living in Rhode Island senior housing demonstrated that increases in fruit and vegetable intake when a mobile produce market selling produce at affordable prices came to the housing sites [20]. This study demonstrates the importance of increasing access to healthier foods for older adults, especially those with mobility difficulties and/or with low income. Therefore, expanding HDMP to include healthy groceries delivered to the home presents an opportunity to enhance nutrition for older adults at nutritional risk.

Adding complementary services beyond meal and food delivery may also be beneficial for recipients of HDMP. One way to enhance HDMP services and their impact on outcomes for older adults is to utilize community health workers (CHWs). Generally, the role of CHWs is based on the community-based workforce, assisting people in overcoming social and financial barriers and, consequently, combating existing disparities [16, 21, 22]. CHWs work to help people connect with an array of services and help prevent health problems, serving as a bridge between individuals and the resources available in the community. Their work is generally focused on low-income individuals and racial/ethnic minorities, which can reduce healthcare inequities [22]. Systematic reviews strongly support the positive impact of the role of CHWs on factors such as depression, loneliness, quality of life, and hospitalizations [15, 17]. A key aspect of CHW support is health coaching. In recent years, robust evidence has demonstrated the effectiveness of CHWs in delivering health information [16]. By including CHWs within organizations that deliver HDMP, CHWs can offer coaching to reduce barriers to healthy eating, provide education and address other social needs faced by older adults.

We describe the protocol for a pragmatic randomized controlled trial in partnership with Meals on Wheels of Rhode Island, Inc. (MOWRI) that aims to test the effectiveness of an enhanced HDMP delivery intervention with two new components - CHW coaching and supplemental grocery bags - on the health and social outcomes of older adults. The trial is rooted in Community-Based Participatory Research (CBPR) with an academic-community partnership between MOWRI and the University of Connecticut researchers evaluating the enhanced HDMP. Research based on CBPR methodology seeks to maintain the subjects of interest at the center of the process, while providing empathy to understand the complex needs and contexts of the individual. CBPR is based on the organization of interdisciplinary teams that allow deeper insights into the sustainability of public health interventions [23].

The CBPR has been shown to strengthen community-research engagement, facilitate understanding of the health issues faced by the community, and identify practical ways to overcome them [24, 25]. Evidence also demonstrates the effectiveness in developing innovative strategies for the older adult population and their specific needs [26–29].

The primary aim of the study is to test whether an enhanced HDMP improves outcomes among older adults. The primary outcome of the study is diet quality. Secondary outcomes include food and nutrition security, loneliness, and health-related quality of life (HRQOL). We hypothesize that clients receiving the enhanced version of the program will demonstrate improvements in these outcomes compared with those receiving only the usual care program, by offering more food, increased social interactions, and an opportunity to connect with additional community resources. This work will advance an understanding of how to increase the impact of HDMP on diet quality and psychosocial outcomes in low-income older adults. This paper represents version 2.0 of the study protocol, finalized on 05/20/2025 (NCT06401694).

## Methods

### Overall study design and population

**Fig. 1:**
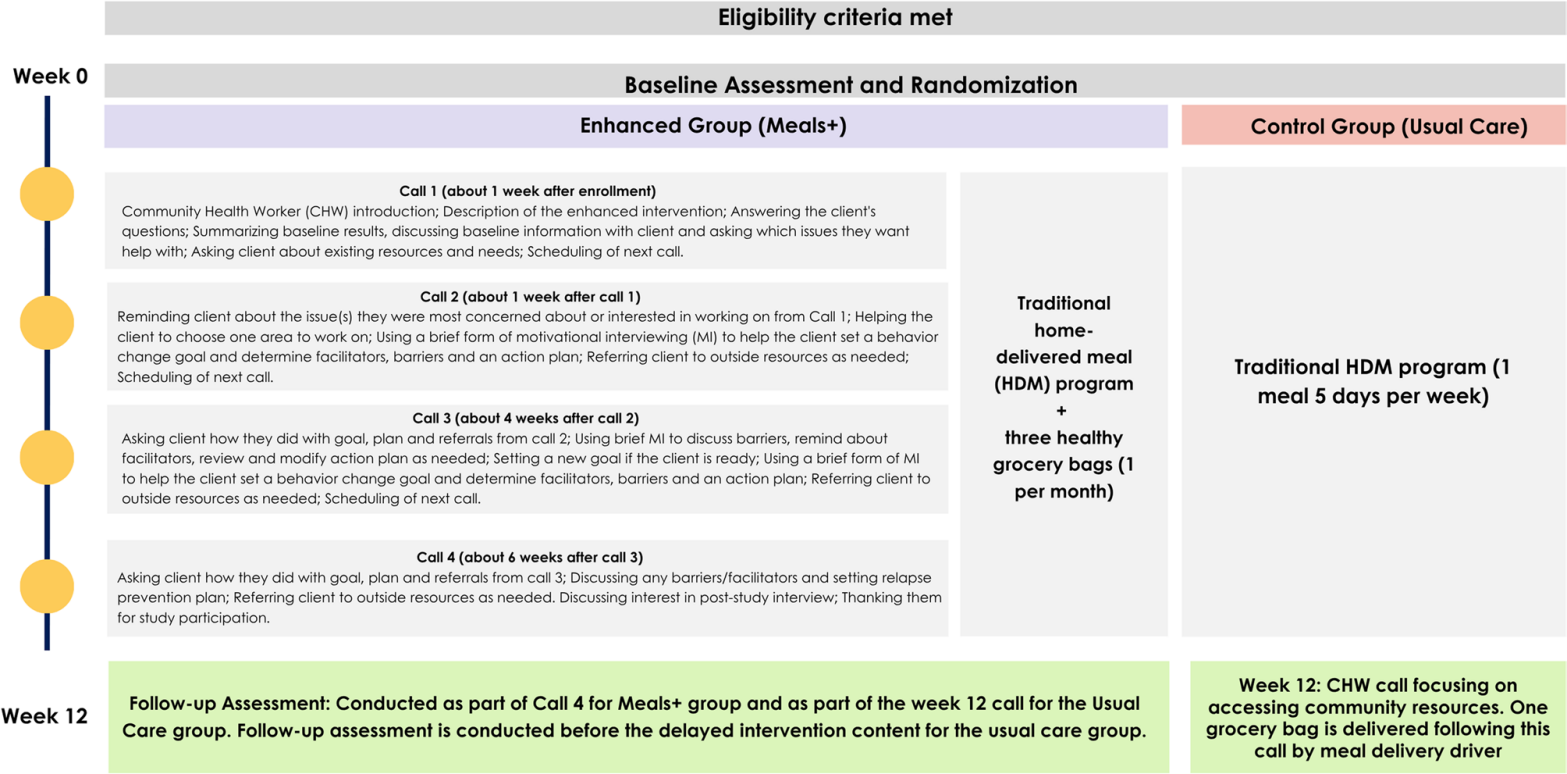
Study Flow Diagram

This is a pragmatic randomized controlled trial with two intervention arms (Fig. 1): a usual care program (home-delivered meals) compared to an enhanced delivery approach (usual care plus grocery bags and CHW coaching calls). All potential MOWRI clients are screened at intake using their usual program eligibility tools including demographics, Nutrition Risk Assessment (NRA) questions, Activities of Daily Living (ADLs), Instrumental Activities of Daily Living (IADLs), living situations, medical conditions, and outside activity. All clients deemed at nutritional risk based on NRA score and meeting other study eligibility criteria undergo a process of informed consent. Those that consent to enroll in the study complete the baseline measures (described below). Then, participants are randomized to usual care or enhanced services (Meals+).

### Eligibility criteria

Study participants are adults 60 years or older who: (1) reside in the state of Rhode Island; (2) can read and speak Spanish or English; (3) are nutritionally at risk as determined by the NRA; (4) are eligible for Title III - funded home-delivered meals assistance. For the pilot study, the NRA score eligibility cutoff was set at ≥ 12. In selecting the NRA cutoff, the study team aimed to enroll participants at high nutritional risk while also considering the expected yield of eligible participants per week given the distribution of clients on the MOWRI waitlist and the team’s capacity to deliver the intervention. This cut-off may be reconsidered based on participant yield in the early stages of the main trial, with the goal of maximizing enrollment while still enrolling participants at high nutritional risk. The exclusion criteria are cognitive or physical limitations that prevent an individual from giving consent or participating in intervention or evaluation activities, as assessed by normal interactions by intake staff or study team at enrollment.

### Ethical considerations and dissemination

The institutional review board (IRB) at the University of Connecticut approved this protocol (BRANY24-073-910). The study underwent an expedited review as it involves no more than minimal risk to the subjects. Given the mode of interactions between participants and the research team, informed consent procedures are administered over the phone with an information sheet sent to the participant. These processes of informed consent approved by the IRB as they were not expected to adversely affect the rights and welfare of the subjects. All study staff (including those at MOWRI) completed the Collaborative Institutional Training Initiative (CITI) Social and Behavioral Research training. This trial is coordinated by the co-principal investigators, with no formal committee. The study protocol follows the 2025 Standard Protocol Items Recommendations for Interventional Trials (SPIRIT) statement [30] (Supplementary File 1).

We will submit all significant protocol modifications to IRB for approval, with modifications to ClinicalTrials.gov if applicable. We will disclose protocol modifications in future publications. We will disseminate results through peer-reviewed publications, formal presentations at international, national and local conferences, and presentations for community stakeholders. Authorship criteria will follow the International Committee of Medical Journal Editors (ICMJE) guidelines [31], with the acknowledgement for contributions for study design development, intervention activities, analysis, and intellectual content review.

### Enrollment and randomization process

Once an eligible client is identified during the initial intake phone call, a CHW gives a brief description of the study and asks if the participant would like to hear more details about the study. Unsure clients are sent information about the study; then the CHW follows up with a phone call as needed. If a client is interested in the study, the CHW verbally walks them through the informed consent script over the phone. If the participant agrees, verbal consent is documented, and a hard copy of the consent information sheet is mailed to the home, if it was not previously mailed. Clients can ask questions at any point during the consent process. After all the clients’ questions have been answered, those who wish to consent indicate that they have understood the study and agree to participate by verbally stating “yes” when asked by the CHW if they would like to enroll. If at any time a client decides that they are not interested in the study, the phone call will conclude, and the client will not be contacted about the study again. However, they can still receive regular home delivered meals from MOWRI.

After participants consent to participate, the CHW conducts the baseline assessment over the phone. This assessment can occur immediately after consent or in a subsequent phone call considering participants’ availability. The baseline assessment includes the following: dietary screening tool (DST), food and nutrition security measures, loneliness and health-related quality of life surveys, demographic questions not included during the routine MOWRI intake process, a health literacy assessment, and an assessment of access to community resources. Participants in both study arms receive a $20 prepaid debit card after completion of the baseline assessment.

Randomization is 1:1, with stratification of participants on living alone vs. living with others. Randomization is performed at the household level and in the case of more than one enrolled client per household, they are randomized to the same study arm. The Research Electronic Data Capture (REDCap) system randomly assigns participants within strata to one of the two conditions after the client completes the baseline survey. The assignment arm is not blinded for study team staff or participants. After randomization, the CHW tells the participant which group they were randomized to and describes the appropriate study arm processes. For participants in the Meals+ intervention group, the CHW describes the upcoming 12-week intervention and asks them to schedule a time for the first CHW coaching session within the next week. For participants in the usual care group, the CHW informs the participant that they will receive the usual MOWRI home-delivered meal service and that they will contact them in about 11 weeks to schedule a time for the 12-week call.

All plans for the design of the intervention and the evaluation were made using a CBPR approach, with decisions made jointly by University of Connecticut researchers and MOWRI leadership and staff, and additional input from community partner stakeholders. The academic-community team met at least weekly to discuss all project-related plans during the development of the intervention and to jointly solve implementation issues that arose during the pilot intervention phase.

## Meals+ Intervention

### Theoretical framework

The intervention is framed by the Socio-Ecological Model (SEM), which highlights the interaction between individuals and community or societal influences, explaining how behavior (i.e. diet) can be affected by multiple levels, including interpersonal influences as well as the home and neighborhood environment and social determinants of health [32]. At the individual level, we have the older adult, with specific personal factors related to nutrition and health-related constraints that can contribute to dietary outcomes, which includes, for example, physical limitations to accessing food and services in the community. Meals+ intervention components that address individual level dietary influences include healthy eating guidance by CHW, as well as recipes and nutrition information provided in the food bags to enhance individual-level knowledge and skills.

At the interpersonal level, CHW and meal-delivery staff can offer social support, resulting in decreased loneliness. CHW can also assist in providing health education and managing health and social needs through connection and coordination of resources at different levels of the SEM, such as food assistance benefits (policy level) and community food resources (community level). Home-delivered meals and food bags reduce barriers to accessing food in community settings. The next level, the organizational level, includes institutions that coordinate and ensure the execution of HDMP. Finally, the policy level includes public policies and programs that finance and sustain HDMP and other food assistance programs influencing the quality of life and ensuring food security for older adults.

The Social Cognitive Theory (SCT) also informs the Meals+ intervention. SCT is an interpersonal level theory that emphasizes a dynamic interaction between people (personal factors), their behavior, and their environments. The CHW interaction generates an important element to develop behavioral capability, and self-efficacy of participants using phone calls to set goals and create action plans to address nutrition, social or health-related issues. Sequential CHW calls provide reinforcement about issues related to the action plan, promoting an iterative process to keep participants on track with their goals and stimulate self-regulation. The food bags provide access to healthy foods, modifying the home food environment and offering recipes and tip sheets. Both experimental intervention elements contribute to improving knowledge about nutrition, attitudes about overall health, skills related to preparing food, and outcome expectations that eating better will result in improved health.

**Fig. 2:**
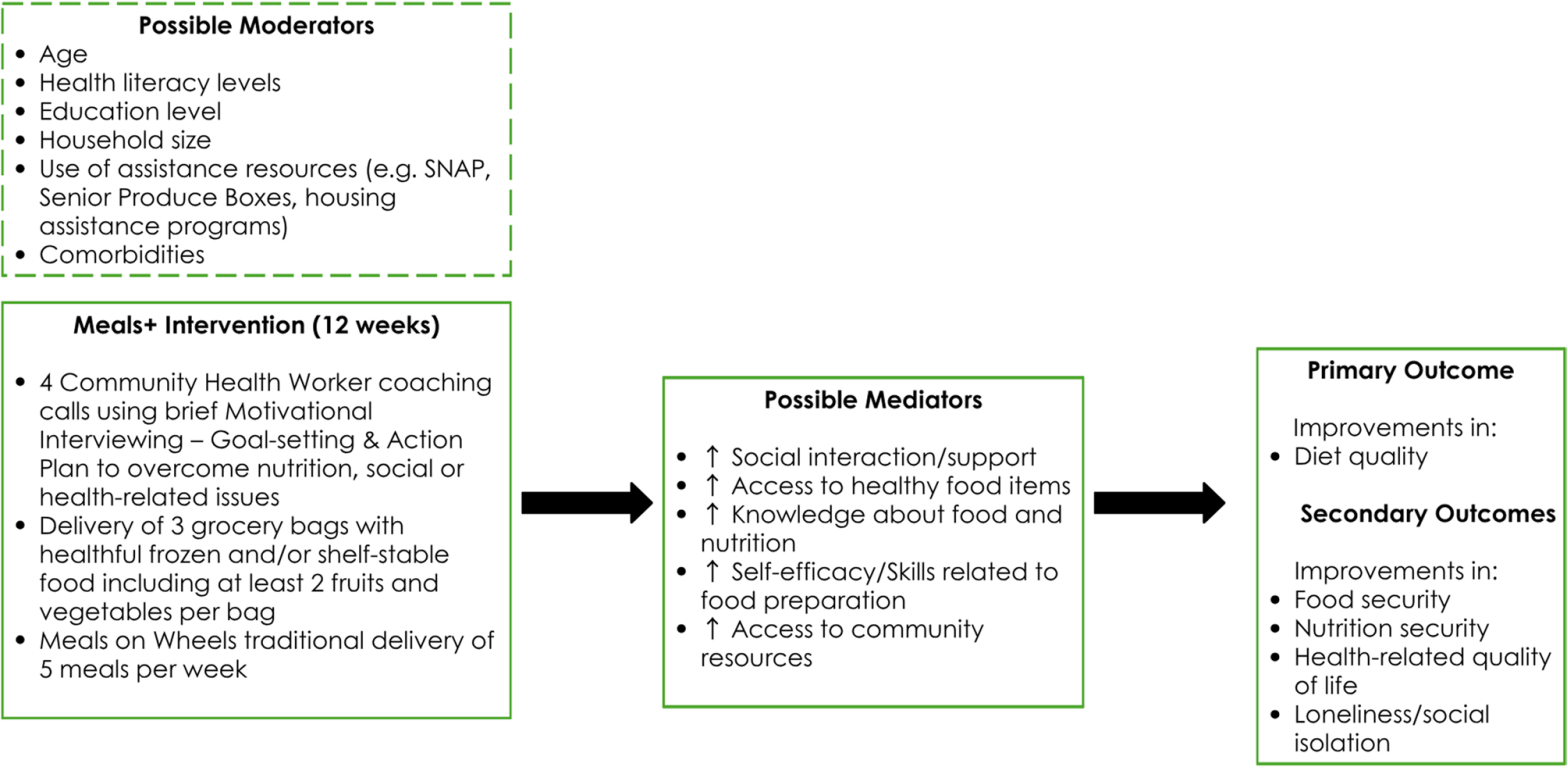
Intervention Logic Model

CHW calls combined with food bag delivery promote a supportive environment to help older adults improve their diet quality and related conditions. Intervention components facilitate improvements in social support and access to healthy food and community services, which can lead to improvements in diet quality. Fig. 2 shows the intervention logic model that explains the interaction between the intervention and expected outcomes. Household size, health literacy level, age, education level and use of assistance resources (e.g. Supplemental Nutrition Assistance Program - SNAP; Senior Produce Boxes; Housing Assistance) are potential moderators related to improvements in diet quality and other outcomes.

### Intervention components– traditional meal delivery

MOWRI provides clients with a prepared meal, delivered to their homes Monday through Friday by a MOWRI driver who also conducts an informal well-being check with a brief social visit. Depending on the area, some clients receive a one-time delivery of five frozen meals. A professional, third-party caterer prepares meals to meet one-third of an older adult’s daily dietary requirements [33]; culturally and medically tailored meals are also available. (Fig. 1).

### Enhanced intervention components

#### CHWs

The first component of the Meals+ intervention is support from a certified CHW. The approach for CHW interactions with clients is to assess needs, identify gaps in care and resources, and then help the client through health coaching and resource coordination. Through these interactions, the CHW addresses social determinants of health, improving food and nutrition security through instrumental and social support, and increasing socialization by offering human connection. The coaching is conducted during four calls, which includes a brief form of motivational interviewing (MI), goal setting, and an action plan focused on addressing some of the client’s nutrition, health, or social issues (Fig. 1). At the final call (Week 12), CHW checks in with the client, and makes referrals to the health insurer or other appropriate community resources for any on-going or unmet needs. Then they conduct follow-up measures (diet quality, food and nutrition security, loneliness, and HRQOL) and ask about satisfaction with the intervention. Fig. 1 describes the content of each call. For any of the four coaching calls, if participants are unresponsive to multiple contact attempts from the CHW, that particular call is considered missed. The participants are contacted during the scheduled time for their next call.

####  Healthy grocery bags

Participants in the enhanced group receive a bag of healthy food once a month for three months. Foods may include, depending on availability, items such as cereal, rice, beans, peanut butter, milk, juice, oats, canned, frozen or fresh fruit, canned, frozen or fresh vegetables, canned poultry, frozen soups, frozen chicken, frozen fish, frozen ground beef. At least two items in each bag will be fruits and vegetables. Foods are provided through the regional food bank, food pantry network, and/or purchased with a discount at a retail store. While the bags are consistent in cost (approximately $75 per bag), the food content of the bags is not the same every month; thus, the bags may vary in terms of calories, macronutrients, or micronutrients. The study team documents and reports each bags’ food and nutrient content. Bags also include recipes and healthy eating guidance materials from different sources such as the SNAP-Ed program, MyPlate [34], the Nutrition and Aging Resource Center [35], and Community Nutrition Education at the University of Rhode Island (URI) [36].

#### Usual care condition

In this study arm, MOWRI provides participants with the traditional HDMP intervention described above that includes five days of prepared meals. After 12 weeks, participants in this group respond to the follow-up assessment and then are provided with one CHW support call as a delayed intervention. This call addresses questions about difficulties finding and accessing community resources and services that participants might need and gives referrals to resources as necessary, including support for filling out applications to internal or external services if needed. Participants in this group are then provided with one grocery bag as part of their next meal delivery.

### Outcomes and measures

#### Primary outcome

The primary outcome of the study is diet quality assessed by the Dietary Screening Tool (DST), which consists of 25 questions focused on dietary intake [37, 38] The DST score ranges from 0 to 100, with an additional 5 points for an affirmative answer regarding the consumption of dietary supplements or vitamins. The questions are based on the consumption frequency of different food groups (e.g. “How often do you usually eat fruit as a snack?”). Scores are treated as continuous variables, and participants are also classified into three groups based on their scores: < 60 “at risk”, 60–75 “possibly at risk”, and > 75 “not at risk”. The DST has been validated for older adults and has adequate sensitivity, specificity, and positive predictive values when compared with the Healthy Eating Index [39, 40].

#### Secondary outcomes

Secondary outcomes include food security, nutrition security, loneliness, and HRQOL. Food security is measured by the United States Department of Agriculture (USDA) 6-item Household Food Security Scale [41]. The scores represent the number of affirmative responses (0–6) with individuals classified in 3 different categories: 0–1 indicates food security, 2–4 indicates low food security, and 5–6 indicates very low food security.

Nutrition security is measured by a 2-item Nutrition Security Screener (NSS) developed by Tufts University, Kaiser Permanente, and Los Angeles Department of Public Health [42]. The NSS includes a short preamble to help with the definition of healthy foods for the respondent, listing examples of foods that support health and well-being. The first question is: “Thinking about the last 3 months, how hard was it for you or your household to regularly get and eat healthy foods?”. The “somewhat hard” respondents are classified as “low nutrition security”, and the “hard” or “very hard” responses are classified as “very low nutrition security.” The “not very hard” or “not hard at all” are classified as “high nutrition security.” The second part of the survey is composed of 13 questions related to the barriers faced by individuals to access healthy foods and are analyzed through binary responses (e.g. “The price of healthy foods limits your ability to eat healthier meals” and “Not having information about which foods are healthy limits your ability to eat healthier meals”).

Subjective isolation or loneliness is measured by the validated University of California, Los Angeles (UCLA) 3-Item Loneliness Scale [43]. The three questions are related to the three dimensions of loneliness (relational connectedness, social connectedness, and self-perceived isolation), with the following content: “How often do you feel that you lack companionship?”; “How often do you feel left out?”; and “How often do you feel isolated from others?”. The score classifies individuals into two different groups: “not lonely” (scores 3 to 5) and “lonely” (scores 6 to 9). The scores of each question are added to generate an individual score.

Health-related quality of life is measured by the Centers for Disease Control and Prevention 4-question measure. The questions explore self-reported general health status (e.g. “Would you say that in general your health is…”) and calculate the number of unhealthy days during the month. The estimation is calculated based on the number of unhealthy physical and mental days. Considering the unhealthy days measure, the participants will be classified into two different groups: “less than 14 days” or “greater than or equal to 14 days” [44].

### Evaluation activities

After the 12-week intervention period, participants in both arms are contacted by CHWs for the follow-up outcome assessment. Participants in the Meals+ arm are asked these questions after the Call 4 intervention component has been completed, while participants in the usual care arm are asked these questions before they receive the delayed intervention call content. For both study arms, the CHW asks about satisfaction with the home-delivered meals and the overall study. For the enhanced study arm, the CHW also asks participants about how much food they consumed from the food bags, use of recipes and educational materials, and satisfaction with the intervention. Participants in both arms receive an additional $30 incentive after completing the 12-week follow-up assessment.

Participants in the Meals+ arm are also asked if they might be interested in participating in a post-intervention satisfaction interview. Of the participants who consent, a subset is selected for interviews, aiming to include participants from all CHWs. UConn staff asks MOWRI for the contact information of those selected participants. Post-intervention interviews ask what participants think about the food bags, recipes, communication with CHW, perspectives about changes related to participation in the intervention (including diet, quality of life and eating habits), and strengths and limitations of the intervention. The participants that complete these interviews receive an additional $25 incentive. These interviews are audio-recorded, and the recordings are sent to a professional transcription company to be transcribed.

The Practical, Robust Implementation and Sustainability Model (PRISM) is the evidence-based framework for evaluation activities [45]. We are measuring PRISM domains using key informant interviews with MOWRI leadership and staff, CHWs and external partners; CHW notes from participant calls; participant evaluation surveys; process evaluation data; MOWRI report data; quality control checks of audiotaped CHW calls; nutritional analysis of food bags; cost tracking; and minutes of project meetings.

### Process evaluation (implementation and sustainability)

Process evaluation measures include: reach (number of clients reached) and representativeness (similar demographic characteristics to the overall MOWRI client population); dose (e.g., percent completion of CHW calls; delivery and use of grocery bags; follow-through on referrals; SNAP enrollment); fidelity (e.g., quality control monitoring of a sample of CHW client calls and post-call forms; content of grocery bags); costs (e.g., CHW time; grocery bags; admin costs); participant satisfaction (e.g. usability, helpfulness, benefits, barriers). Regarding fidelity, a subset of audio-recordings of the Meals+ CHW/participant calls are automatically transcribed by the MOWRI Ring Sense, a product by RingCentral computer system. The system also translates the calls with Spanish speakers’ participants. Then the transcripts are reviewed for fidelity to the intended call content by the study team using a checklist. We are also measuring overall retention and by CHW. If issues are found, retraining and/or meetings will be held with the CHWs to improve fidelity to the call protocol. For the grocery bags, we will analyze the foods and nutrient content of the foods provided in each month’s bag based on the 2020–2025 Dietary Guidelines for Americans and MyPlate recommendations for food servings, macronutrients and micronutrients per month and per day [34, 46]. This data will be communicated to MOWRI and adjustments to the food bags will be made if necessary.

Ongoing stakeholder interviews will provide data regarding context, barriers, facilitators, usability, and burden, which will help us to measure the sustainability potential. By participating in interviews, these stakeholders provide insights about key characteristics of existing MOWRI program and clients, current program operations, internal and external communication, and organizational perspective on the integration of enhanced intervention into traditional activities, evaluating the alignment with MOWRI mission and potential concern about unanticipated effects. In addition, the Program Sustainability Assessment Tool [47] is administered to MOWRI leadership, CHW, and key stakeholders to collect quantitative results about potential sustainability of the enhanced intervention. We will analyze process data continually during the implementation phase and discuss it during project meetings to determine possible adaptations or modifications.

### Sample size and power calculations

We conducted a power analysis for this randomized controlled trial (RCT) using Statistical Analysis System (SAS) version 9.4 (Cary, NC). Assuming that the standard deviation for the primary outcome, DST, is 12.0 [48], and a significance level of $\alpha = 0.05$ , we will have 80.6% power to detect a difference between the arms of 2.0 points on the DST using 574 participants in each study arm. Because we are expecting 70% retention in each arm we will enroll 1640 participants in the study. We performed this sample size calculation based on assumptions from the literature.

The study uses an intent-to-treat approach so that participants who decline MOWRI program or intervention activities (i.e., deciding not to receive weekly meals from MOWRI, participate in the CHW phone calls, or receive the monthly food bags), remain in the study and will be analyzed according to their assigned intervention. Participants can stop receiving MOWRI services and still receive a follow-up call and continue to be part of the study until follow-up. Participants who are no longer getting home-delivered meals will no longer get the enhanced program of CHW calls or food bags. Participants will be withdrawn from the study if either: (1) they verbally disclose that they no longer wish to participate in the research; (2) they have not had any calls with the CHW by week 8; or (3) if they are in an institutional setting (e.g., hospitalization or inpatient rehabilitation) at the time the follow-up assessment is scheduled to occur, and this stay lasts > 14 days. In addition to the intention-to-treat analyses, we will examine the effects of the intervention among participants who would have complied with and adhered to the assigned intervention using principal stratification analysis [49, 50].

### Data monitoring

The researchers keep all study data on a secure server with restricted access. Research records are coded with numeric identification codes stored separately from participant identifiable information collected by MOWRI as part of its normal programming. All data extracted from secure databases are stored in cloud-based institutional folders accessible to members of the study team. Any computer hosting such files has password protection to prevent unauthorized access. Only the members of the research staff have access to the data. All calls with participants are completed in a private area with a headset.

In terms of the audit process, we will generate reports at scheduled intervals. We will share and review reports as a standing agenda item at the weekly study team meetings. Adverse events, serious adverse events, and unanticipated problems may be identified by any member of the research or intervention team. They will be entered into REDCap and notifications will be sent to the study principal investigators and project coordinator within 24 h.

### Data analysis

We will use quantitative variables to describe the general characteristics of the population, presenting the mean or standard deviation or median and interquartile range for continuous variables and frequency for categorical variables.

To compare the differences in the effects of Meals+ and usual care on DST, we will implement a hierarchical model to account for the correlation between the baseline and follow-up assessments. Formally, let $DSTij$ be the primary outcome for participant $i\in\left\{1,...,n\right\}$ at timepoint $j\in\left\{\mathrm{baseline},\;\mathrm{follow}-\mathrm{up}\right\}\;$ , $W_i\in\left\{\mathrm{Meals}+,\mathrm{usual}-\mathrm{care}\right\}$ the intervention assignment for participant ${i}$, and $\mathrm{X}_i$ be a set of baseline covariates for participant ${i}$, we will assume that $DST_{ij}\mid X_i,W_i=\beta_0+\gamma_j+\delta_1W_i+\delta_2W_i\,I(j=\text{Meals+})+\beta X_i+\varphi_i+\epsilon_{ij}$, where $\beta_0$ is the conditional average for the usual-care arm at baseline, $\gamma_j$ is timepoint effect for the usual-care arm, $\delta_1$ is the difference between the Meals+ arm and the usual-care arm at baseline, $\delta_2$ is the difference between the two arms at follow-up, $\beta$ are covariates effect $\varphi_i\sim\mathcal N(0,\sigma_\varphi)$ is subject-specific effect, and $\epsilon_{ij} \sim \mathcal{N}(0, \sigma_{\epsilon})$ are random errors. Using this model, we will estimate the marginal difference between the two arms at follow-up. For the secondary outcomes we will use similar generalized linear models depending on the distribution and nature of the outcome variables. We will summarize baseline characteristics of participants by arm and whether their outcome data is missing. To address missing values, we will implement a multiple imputation procedure [51, 52].

Principal stratification analysis will rely on a Bayesian framework to estimate the compliers average causal effect [50, 53]. Bayesian methodology allows us to examine the sensitivity of the estimates to commonly-used identifying assumptions (e.g., monotonicity, exclusion restriction). In addition, this analysis can identify participants’ characteristics that are correlated with intervention compliance and adherence, which can lead for tailoring of future interventions.

Lastly, as an exploratory analysis we will examine the heterogeneity of treatment effects by including an interaction term between the intervention indicator, timepoint and a subgroup indicator in the hierarchical regression models. Among factors that will be examined would be baseline loneliness, nutrition security, food security, HRQOL, and other demographic characteristics.

### Study modifications following pilot intervention

The study protocol described above introduces several Changes because of observations made during a pilot study with 12 participants from July to October of 2024. Considering the eligibility process, the pilot study did not allow us to test recruitment and intervention at full capacity; therefore, we did not change the NRA score eligibility cutoff before initiation of the full-scale RCT. Inclusion of participants with a lower NRA score may still be considered if new information emerges about the appropriateness of this cutoff for enrolling clients at high nutritional risk while also considering the yield of participants and the team’s capacity to deliver the intervention. For recruitment, we added a mailing after the initial intake call for participants who were unsure about study participation. This mailing from MOWRI includes information about the study as well as a copy of the consent information sheet.

Calls for intervention participants were initially limited to one call per event, with a limit of four calls at maximum. We removed this limitation, as we found that sometimes more than one call was needed to reach participants or to continue a previous conversation at a time when the participant was better able to focus on specific call topics, instead of redirecting the conversation to other topics. This change also reduces barriers to communication and the burden of time.

We revised certain questions to minimize participant confusion. Prompted by participant and CHW feedback, we changed the measure of nutrition security from the Nutrition Security, Healthfulness Choice and Dietary Choice [54] measure to the NSS measure as some of the questions on the original tool were unclear or sounded repetitive to participants. We also rephrased some questions on the Meals+ CHW Call 4 satisfaction questionnaire to increase comprehension. All the language adaptations aim to facilitate communication between CHWs and participants. We made some modifications to the CHW coaching call outlines to provide conversation anchors that focused on the discussions about study outcome related issues.

## Discussion

Home-delivered meal programs help to reduce nutritional risk and promote socialization among older adults. However, evidence shows that older adults still face challenges to address nutritional, social, and health needs [8–11, 19]. This pragmatic randomized controlled trial will examine the efficacy of an enhanced HDMP that includes CHW coaching and healthy grocery bag delivery on improving diet quality, food and nutrition security, loneliness and HRQOL in older adults. By comparing the outcomes of participants receiving enhanced services with those receiving usual services, the study will provide evidence on the additional benefits of incorporating innovative elements into the traditional HDMP.

The study has a rigorous randomized design. This ensures that the differences observed between the groups after the intervention are attributable to the intervention and not to other factors. The study is based on the CBPR approach, with the active contribution of MOWRI and community stakeholders in intervention development and implementation. Conducting the intervention in real world setting of MOWRI increases external validity, increasing the likelihood that the intervention enhancements can be generalized to other HDMP. The innovative additions of healthy grocery bags and CHW coaching will be important in the light of new opportunities for Medicaid reimbursement for services, including providing groceries for people with diet-related conditions or food insecurity and the “Food is Medicine” expansion [55].

This trial has some potential limitations. First, we will not have the ability to assess separate effects of each component of this multi-component intervention. However, interventions with multiple components can maximize the impact of the intervention on the target population, which might not be possible with just one component alone. Furthermore, the combination of components mimics the normal public health scenario, which works with several variables to address health problems [56, 57]. Beyond that, with our evaluation process, we could assess satisfaction with each intervention component and examine the relationship between intervention component delivery and outcomes.

Another potential challenge is that older adults may have barriers to intervention adherence, including multiple clinical conditions, hospitalizations, minor cognitive and memory problems, and social isolation. Older adults at the highest nutritional risk may disproportionately experience these barriers. We currently enroll participants with an NRA score *≥* 12, but the standard of practice is that older adults with an NRA score of 6 or greater are considered high nutritional risk [58, 59]. Thus, we may need to be more inclusive to reach an adequate sample of participants. At the same time, we may find that the highest risk older adults are also the most reluctant to participate in a research study. Additional efforts to bolster enrollment may be needed, including a potential expansion of study eligibility to include participants with NRA scores lower than 12, but still at high nutritional risk.

Although our aim is to identify significant differences between the intervention and the control groups, it is important to consider that, even in the absence of significant differences between groups, HDMP will continue to operate and provide known benefits to the health and quality of life of older adults at nutritional risk. Therefore, this study will also focus on expanding the understanding of the administrative and operational processes of the program, which includes the evaluation of implementation and sustainability elements such as cost of food bags and CHW time, participant satisfaction, program retention, intervention fidelity, and stakeholders’ perspectives about the intervention and its sustainability. Study findings, including outcome and process evaluation, may guide future improvements in HDMP practices, with potential benefits in access to food, community services and social support for older adults in need.

## Conclusion

We describe the intervention and evaluation protocols for a randomized controlled trial to compare Meals+ to usual care home-delivered meal program. This study represents an innovative strategy to achieve nutritional and social goals for older adults with vulnerabilities, seeking to improve diet quality, access to healthy food, and overcome limitations in social determinants of health.

## Supplementary Information

Supplementary Material 1.

## Data Availability

No datasets were generated or analysed during the current study.

## Abbreviations

ADLs: Activities of Daily Living
CBPR: Community-Based Participatory Research
CHWs: Community health workers
CHW: Community health worker
CITI: Collaborative Institutional Training Initiative
Co-PIs: Co-principal Investigator
DST: Dietary screening tool
HDMP: Home-delivered meal programs
HEI: Healthy Eating Index
HRQOL: Health-related quality of life
IADLs: Instrumental activities of daily living
ICMJE: International committee of medical journal editors
IRB: Institutional review board
MI: Motivational interviewing
MOWRI: Meals on Wheels of Rhode Island, Inc.
NRA: Nutritional risk assessment
NSS: Nutrition security screener
PRISM: Practical, Robust Implementation and Sustainability Model
RCT: Randomized controlled trial
REDCap: Research electronic data capture
SAS: Statistical analysis system
SCT: Social Cognitive Theory
SEM: Socio-Ecological Model
SNAP: Supplemental nutrition assistance program
SPIRIT: Standard protocol items recommendations for interventional trials
UCLA: University of California, Los Angeles
URI: University of Rhode Island
USDA: United States Department of Agriculture
